# A discrete choice experiment to elicit preferences for a liver screening programme in Queensland, Australia: a mixed methods study to select attributes and levels

**DOI:** 10.1186/s12913-023-09934-2

**Published:** 2023-09-05

**Authors:** Michelle J Allen, Rachael Doran, David Brain, Elizabeth E Powell, James O’Beirne, Patricia C Valery, Adrian Barnett, Ruvini Hettiarachchi, Ingrid J Hickman, Sanjeewa Kularatna

**Affiliations:** 1 Australian Centre for Health Services Innovation and Centre for Healthcare Transformation, School of Public health and Social Work, Faculty of Health, Queensland University of Technology, Brisbane, QLD Australia; 2 Department of Economics and Related Studies, University of York, York, UK; 3 The University of Queensland, St Lucia, QLD Australia; 4 Centre for Liver Disease Research, Translational Research Institute, Faculty of Medicine, The University of Queensland, Woolloongabba, QLD Australia; 5 Department of Gastroenterology and Hepatology, Princess Alexandra Hospital, Woolloongabba, QLD Australia; 6 University of the Sunshine Coast, Maroochydore DC, QLD Australia; 7 Sunshine Coast University Hospital, Birtinya, QLD Australia; 8 QIMR Berghofer Medical Research Institute, Herston, QLD Australia; 9 Department of Nutrition and Dietetics, Princess Alexandra Hospital, Woolloongabba, QLD Australia

**Keywords:** Non-alcoholic fatty liver disease, Attribute Development, Discrete choice experiment, Patient preferences, Community Screening

## Abstract

**Background:**

In Australia, the overall prevalence of liver disease is increasing. Maximising uptake of community screening programmes by understanding patient preferences is integral to developing consumer-centred care models for liver disease. Discrete choice experiments (DCEs) are widely used to elicit preferences for various healthcare services. Attribute development is a vital component of a well-designed DCE and should be described in sufficient detail for others to assess the validity of outcomes. Hence, this study aimed to create a list of potential attributes and levels which can be used in a DCE study to elicit preferences for chronic liver disease screening programmes.

**Methods:**

Key attributes were developed through a multi-stage, mixed methods design. Focus groups were held with consumers and health care providers on attributes of community screening programmes for liver disease. Stakeholders then prioritised attributes generated from the focus group in order of importance via an online prioritisation survey. The outcomes of the prioritisation exercise were then reviewed and refined by an expert panel to ensure clinically meaningful levels and relevance for a DCE survey.

**Results:**

Fifteen attributes were generated during the focus group sessions deemed necessary to design liver disease screening services. Outcomes of the prioritisation exercise and expert panel stages recognised five attributes, with three levels each, for inclusion in a DCE survey to elicit consumer preferences for community screening for liver disease. This study also highlights broader social issues such as the stigma around liver disease that require careful consideration by policy makers when designing or implementing a liver screening programme.

**Conclusions:**

The attributes and levels identified will inform future DCE surveys to understand consumer preferences for community screening programmes for liver disease. In addition, the outcomes will help inform the implementation of the LOCATE-NAFLD programme in real-world practice, and could be relevant for other liver and non-liver related chronic disease screening programmes.

**Supplementary Information:**

The online version contains supplementary material available at 10.1186/s12913-023-09934-2.

## Background

Liver disease involves a large range of conditions including viral hepatitis, alcohol related, and non-alcohol related fatty liver disease, autoimmune conditions, and liver cancer [1]. Non-alcoholic fatty liver disease (NAFLD) is a spectrum of conditions characterised by excessive fat deposition in the liver in association with metabolic dysfunction [2, 3]. It is the most common chronic liver disease in the world [4] and its burden is felt in Australia where the number of prevalent cases is expected to grow from 5.5 million to 7 million by 2030, in line with expected population growth [5]. NAFLD is often accompanied by metabolic risk factors such as obesity, hypertension, and type 2 diabetes mellitus, as well as possible genetic factors [6, 7]. A subgroup of people with NAFLD develop a necroinflammatory subtype, non-alcoholic steatohepatitis, with liver cell injury and inflammation. Some of these patients may develop progressive fibrosis that can lead to cirrhosis and related complications such as hepatic encephalopathy and hepatocellular carcinoma. Due to shared cardiometabolic risk factors, extrahepatic conditions including cardiovascular disease, extrahepatic malignancy and chronic kidney disease are common comorbidities [8–15].

Of concern, cases of advanced-stage fibrosis are predicted to increase by 50–70% by 2030, with decompensated cirrhosis, primary liver cancers and liver transplants predicted to increase by 85% and overall mortality in the NAFLD population by around 55% [16]. In 2012, the Australian Institute of Health and Welfare reported that much of the $5.4 billion spent annually on liver disease could be attributed to NAFLD, demonstrating a substantial cost burden [17]. The impact due to loss of productivity was estimated at around $4.2 billion, with $2.1 billion associated with lower employment rates and $207 million from absenteeism, driven by those with advanced disease [17]. Nearly $450 million was spent on hospital admissions, out-of-hospital services and prescription medication, and informal care expenditure also reached $260 million [17]. As well as the enormous cost burden, patient quality of life (QoL) has been severely reduced for those with advanced disease [18].

NAFLD is often detected incidentally via altered liver biochemistry or during investigations for other medical issues [19, 20]. It is estimated that up to 17% of patients with NAFLD are not referred for specialist secondary care when needed, leading to a higher risk of future disease complications, hospital admission and the inevitable high costs [21–24]. On the opposite end of the spectrum, 80 to 90% of patients are not at risk of advanced liver disease and can be appropriately managed in primary care. This highlights a need for a screening tool that facilitates the timely management of NAFLD and prevents referral decisions that lead to unnecessary costly investigational tests, and complex management plans [5]. Screening programmes can be effective in the early identification of advanced fibrosis, enabling intensive management while there is opportunity for reversal of steatosis/fibrosis and early liver cancer surveillance, which increase the applicability and cost-effectiveness of hepatocellular carcinoma therapies [25]. Multiple screening tests are available for assessing the extent of liver disease that range in effectiveness, cost, and invasiveness, including liver biopsy, blood tests (such as FIB-4 and ELF), and transient elastography.

In Australia, a new model, the LOCal Assessment and Triage Evaluation of Non-Alcoholic Fatty Liver Disease (LOCATE-NAFLD) has been designed to support the identification of early stages of NAFLD by clinicians. This new model has been designed to improve efficiency in disease management and to alleviate the clinical, consumer, and economic burden of NAFLD [5]. The model uses transient elastography, a specialised, painless and non-invasive ultrasound procedure that assesses the extent of hepatic fibrosis [26], that has been shown to be effective in early diagnosis of advanced fibrosis among high-risk populations, such as patients with type 2 diabetes [22]. Future widespread implementation of such a model of care largely depends on consumer acceptance and service usage, especially as NAFLD is an asymptomatic disease at the initial stage. Hence, successfully delivering this care model ultimately depends on alignment with consumer preferences.

A discrete choice experiment (DCE) is an evaluation technique that can be used to study the preferences of healthcare consumers and providers [27], it has become the most frequently applied approach in health care in recent years [28]. While the use of DCEs in healthcare is increasing, only a few have been designed to elicit preferences for a community-based health screening programme, with the literature particularly bare regarding liver disease [29]. Appropriately developed, designed, conducted, analysed and interpreted DCEs can offer numerous benefits to the health sector. Most importantly, they provide rich data for economic evaluation and decision-making [30]. In general, a DCE is used to understand which characteristics or attributes are preferred by consumers, how they balance them, and the relative importance of each attribute in their decision to consume [31]. Identifying and prioritising key attributes and levels based on consumers’ views is vital to developing a DCE survey, but are rarely published or articulated in enough detail for replication [32]. Hence, this study aimed to explore potential attributes and levels that can be used in a DCE study to elicit preferences for community-based health screening programmes for liver disease. The outcomes of this DCE will help inform the implementation of the LOCATE-NAFLD programme in real-world practice and could be relevant for other liver and non-liver related chronic disease screening programmes. This paper details the attribute development for the DCE, with subsequent DCE stages and broader trial results reported elsewhere.

## Methods

**Fig. 1:**
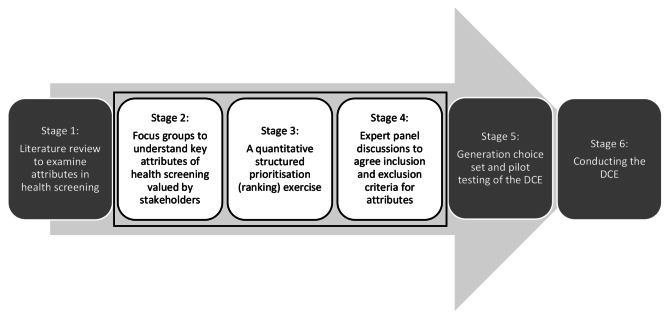
Summary of six-step study designed to elicit preferences for health screening services for chronic disease

We used a multi-stage process (Fig. 1) - developed by De Brun et al. [32] to explore consumer and expert opinions and perceptions of what constitutes an effective health screening programme and how different attributes impact service delivery and overall success. A systematic review of published DCEs (Stage 1, Fig. 1) designed to elicit stakeholder preferences for community-based health screening programmes has shown that preferences are contingent on the specific context of the programme [29]. Overall findings of the literature demonstrated 58 attributes of interest across the 27 studies, highlighting a wide variation in preferences (Additional File 1). The focus of this paper is attribute development which encompasses three stages - Stage 2: identifying the key attributes by conducting focus groups with key stakeholders; Stage 3: identifying the essential attributes by conducting a quantitative structured online prioritisation exercise; and Stage 4: identifying potential attributes and levels for potential use in a discrete choice experiment by conducting an expert panel discussion. Ethics approval for this study was obtained from the Queensland University of Technology Human Research Ethics Committee (approval number HREA 2022–4282) and all methods were performed in accordance with the relevant guidelines and regulations. Stages 5 and 6 of the study, the pilot testing and conduct of the DCE survey, will be published in further work.

### Focus groups (stage 2)

Focus groups were conducted via an online teleconferencing platform facilitated by an experienced qualitative and mixed methods researcher (MA) to understand the key attributes. The targeted sample size was between six to twelve participants, chosen as a balance between pragmatic considerations including the range of attributes already available from the literature, cost and time, and established guidance on the conduct of focus groups [33, 34]. Participants were purposively sampled to ensure a variety of clinicians and researchers who work across Hepatology in the community and hospital sectors and experienced patient representatives/ advocates with lived experience of liver disease and research.

Potential participants were identified through the chief investigators’ existing contacts and professional networks, and via Hepatitis Queensland, an advocacy and support service for hepatitis and liver disease. Initial invitations were sent via email along with a participant information sheet and consent form, for which a signature was required prior to attending the focus group meeting. A semi-structured interview guide was developed (Additional File 2), based on attributes identified in the literature review [29], and was used by the group facilitator to guide focus group discussions. The document was designed flexibly, such that relevant themes raised during the sessions could be followed up and explored further.

All focus groups lasted between 51 and 89 min. Additional notes were recorded by the researcher (MA) and two other analysts (RD, AJ) that attended the meetings. Author MA is a qualitative and mixed methods researcher specialising in implementation science, health service design and evaluation, and is experienced in facilitating interviews and focus group discussions. RD was undertaking a Masters in Health Economics, of which this study (stages 2 to 4) is a part of her dissertation. AJ is a health economist who led the systematic review (Stage 1). A summary of findings was discussed at the end of each focus group session for comment by participants. Before coding, all contributions were digitally audio recorded and professionally transcribed. Anonymised transcripts were analysed independently by two researchers (MA, RD). A predominantly deductive thematic analysis technique approach was used for qualitative analysis. The codebook, with attributes drawn from the literature, was used as a starting point to organise the themes and sub-themes relating to attributes that are considered important in influencing consumer uptake of chronic disease screening programmes and, therefore, would be valuable to implement into the DCE choice sets. This involved identifying key data points and themes systematically to gain a preliminary understanding of participant perspectives on a given topic [35, 36]. Direct quotes from focus group participants that were associated with different attributes and levels were organised using MS Excel. Summaries of the data were also drawn up to highlight themes from interview transcripts. Any additional themes that did not fit the initial codebook were added, including several barriers and facilitators relevant to implementing community screening programmes.

### Structured prioritisation exercise (stage 3)

Following identification of relevant attributes from focus group participants, a structured prioritisation exercise was conducted to examine the relative importance of each attribute. The structured prioritisation exercise was conducted as an online survey in which participants ranked attributes from ‘most’ to ‘least’ important, with the data submitted using Qualtrics, a specialist survey software. Participants were recruited using the same process as the focus groups.

The relative ranking of attributes was analysed across all participants and then separately for consumers and healthcare providers to understand any participant-group-related differences. To produce a summary score, the ranked attributes were scored based on their assigned rank and the mean score was calculated for each attribute. The attribute list was sorted in ascending order, and the attribute that produced the lowest mean score was considered the most important, while the attribute with the highest mean score was considered the least important. Attributes deemed of greater importance formed the basis of discussion and finalisation by the expert panel in Stage 4: expert panel discussion.

### Expert panel (stage 4)

The expert panel (n = 6) was a multidisciplinary group including health economists, scientific researchers, and clinician-researchers with expertise in liver disease. Expert panel discussion was conducted via an online teleconferencing platform. To establish the most important attributes that would form the final DCE choice set, the panel discussed the three ranked attributes lists (non-blinded):

(i) consumer and healthcare provider combined,
(ii) consumer only, and.
(iii) healthcare provider only.

When deciding which attributes to include in the DCE survey, the panel considered multiple factors. These factors included: input from stakeholders about the relative importance of attributes (results from Stage 3); relevance of attributes to the goal of the DCE survey (eliciting patient preferences for different liver disease screening options); and attribute relationships to each other [37, 38].

Once the attributes were agreed upon, each attribute’s levels were discussed. Levels are the different options available within each attribute, for example if an attribute was ‘mode of delivery’, the levels might be ‘face to face’, ‘videoconference’, and ‘telephone’. After the discussion, meeting notes and the proposed attribute list with levels were reviewed, refined, and finalised via email with all panel participants and chief investigators.

## Results

### Focus groups (stage 2)

Seven participants, across three sessions, were asked about existing community-based health screening programmes, the current attributes of those programmes, and their preferences relating to an ideal screening programme. A further five participants (3 clinicians and 2 consumers) were invited to participate but were unable to attend (response rate − 7 out of 12, 58.3%). The sample was considered enough to gather potential attributes given the mix of clinicians and lived experience of the consumers. Participants in the focus groups were (a) Chronic disease clinician-researchers from the University of the Sunshine Coast, University of Queensland and QIMR Berghofer Medical Research Institute (QIMR-B) (n = 3); (b) community-based nurses who are involved in community health screening programmes in the Sunshine Coast and Metro South Primary Health Network regions (n = 2); and (c) healthcare consumers with chronic conditions such as diabetes, cardiovascular and liver disease, with extensive experience in advocacy and research (n = 2). Participants from each group are in Table 1.

**Table 1:** Details of focus group participants

Focus Group	Participants (n)	Participant type and background	Gender
Group 1	2	• Clinician - specialist (hepatology) • Clinician - nurse (hepatology)	M F
Group 2	2	• Clinician/ researcher (hepatology) • Clinician/ researcher (chronic disease, incl. hepatology and cancer)	F F
Group 3	3	• Consumer and patient advocate (chronic disease and Aboriginal and Torres Strait Islander health) • Consumer and patient advocate (hepatology) • Clinician - nurse (hepatology)	F M F

Qualitative analysis of the transcribed focus group interviews identified several potential attributes and levels that are viewed as important in maximising uptake of screening programmes. Based on the hepatology backgrounds and experience of the participants (both clinicians and consumers), much of the discussion focussed on chronic liver disease such as Hepatitis and NAFLD related screening options.

Analysis revealed 15 attributes that participants perceived to be policy-relevant in liver disease screening (Table 2). The focus group discussions coalesced under five themes:

**Table 2:** Attributes and themes from focus group discussions

Theme	Attribute of screening program
Convenience and ease of access	Patient receives a reminder or prompt to undertake screening
	Ease of making an appointment to be screened
	Travel distance to screening location
	Out of pocket costs for the patient
	Screening is integrated into a routine care appointment
Health worker and consumer interaction	Positive patient experience with staff (e.g. friendly, culturally safe, and non-judgemental)
	Information on screening process and/or value of being screened comes from a trusted source
Consumer motivation	Patients experience of pain/discomfort during procedure
	Severity of the condition, current symptoms, and a patient’s other co-morbidities/conditions
	Likelihood that additional testing or invasive testing is required
	Availability and effectiveness of treatment options
Screening test and process	Waiting time for results
	Quality of the test and results (e.g. accuracy, consistency)
System level	Screening data is part of a registry to inform population health decisions e.g. where to put more services
	Staff are trained and knowledgeable about the condition and screening process

1. Convenience and ease of access.
2. Consumer and healthcare worker interactions.
3. Consumer motivation.
4. Screening test and process.
5. System level factors.

In addition, barriers and other implementation related information that came from the focus group discussions have been reported to give those who design and implement screening services some broader context, and to honour the useful information that was provided by participants.

The first theme relates to the convenience or ease of accessing screening. Attributes such as the distance (or time) to travel to the screening location and ease of making an appointment were highlighted often throughout the focus group sessions as key to maximising uptake.

*“Easy to do. Easy to book. Easy to park… There’s no cost involved… Make it easy for them to take the option to do it rather than not do it.“ Clinician3*.

*“When you live quite a distance … you’ve got to take a bus and a boat and a bus and a train and a walk to get to the hospital.” Consumer1*.

Additional features to increase convenience such as reminders for consumers or providing the screening as part of a standard health visit were also discussed and added as attributes for consideration.

*“I think setting’s important… perform screening for advanced fibrosis in healthcare settings where people are already engaged… there’s definitely a convenience factor… 100%, yeah.“ Clinican1*.

*“And patients could get reminders. Like I get a reminder for my cervical cancer screening, breast screening.“ Clinician4*.

Out-of-pocket costs to consumers was also noted as a key attribute which should be considered when designing a screening programme. Out of pocket costs were deemed to include not only the screening test, but the additional costs such as parking, and time off work borne by the consumer.

*“People don’t like travelling and certainly don’t like paying parking fees at hospitals and all the things like that. So, the cheaper and more local it is (the better).“ Clinician1*.

*“If there’s a cost involved then that’s going to be, okay… it’s going to be a toss-up between do I pay my bills and buy food, or do I do this test?“ Consumer1*.

The way in which consumers and healthcare workers interacted in relation to the screening process was another theme of the focus group discussion (Theme 2).

Participants noted that a positive consumer experience (with staff) was an attribute important to the likelihood of screening uptake. Participants expressed a level of stigma associated with liver conditions, which needed to be addressed in future screening programmes.

*“I was getting put into a basket, into a pigeonhole, treating us all like we were drinkers or drug users, and there’d be a lot of people who can relate to that.“ Consumer2*.

*“Possibly the factors that often lead to liver disease… So, alcohol and obesity and illicit drug use… for too long it’s perhaps been seen in a negative light.“ Clinician4*.

It was decided that an attribute for positive consumer experience should specifically mention non-judgemental attitudes (to address stigma) and culturally safe interactions for Aboriginal and Torres Strait Islander peoples.

*“So that really welcoming environment where it’s culturally appropriate and safe, and non-judgmental is a really key component of making it – you want to come in and see the service.“ Consumer2*.

*“Having the health staff that are doing the test, culturally aware, for our mob.“ Consumer1*.

Participants also noted that healthcare and consumer interactions begin before the screening visit takes place. Due to a current lack of awareness about liver disease or the need for screening, the communication of this information was also considered important for acceptability of a screening programme. The source of screening information, in particular the level of trust, was added as another attribute for consideration.

*“I just don’t see much awareness around it (liver disease) …where I live anyway, yeah.“ Consumer2*.

*“So, it was a trusted source of information and that sort of encouraging from someone that they know …removes that sort of fear of the unknown.“ Clinician2*.

Several participants suggested that lived experience advocates and community leaders are often considered trustworthy information sources and could be used as a potential mechanism to increase awareness in a community.

*“Still believe it comes down to working with what we have in the communities, work with leaders, leaders of communities, because that’s where you get the respect.“ Consumer2*.

*“Some of the patients said that, if only I knew somebody that had already gone through this, they could have told me what to expect or even just to know that there was someone else out there that’s going through the same thing.“ Consumer1*.

*“We had people like (Rugby player 1) supporting us, (Rugby player 2) was supporting us… I reckon with the awareness around anything – it can be fatty liver disease, hepatitis, whatever, you’ve got to have Community Champions out there.“ Consumer2*.

Consumer motivation for screening was explored in the third theme. Pain or physical discomfort experienced during screening was noted to have a big impact on whether engage in a screening programme, so was included as an attribute for consideration.

*“It doesn’t hurt; it doesn’t scare people.“ Clinician3*.

*“Mainly physical. I mean, you know, people won’t like to turn up at colonoscopies and endoscopies because it’s uncomfortable… for something like a blood pressure or a Fibroscan, it doesn’t even involve the discomfort of a blood test… it’s an ideal test in that regard.“ Clinican1*.

Another attribute discussed was the impact of a consumer’s current physical condition on their motivation to undertake screening in terms how severe their symptoms were, as well as their other co-morbidities they might have.

*“I think symptoms play a part as well. You know, this is – people don’t often know they’ve got NAFLD or Hep C or Hep B until something presents, and so it’s, you know, “Who cares?“ Clinician2*.

*“They’re already dealing with a certain amount of comorbidity, and, I guess, actively undergoing a test that might lead to another diagnosis is – probably puts them off a little bit.“ Clinician2*.

Another attribute in this theme was the likelihood or risk associated with further testing or invasive testing.

*“This is one of the problems associated with liver disease… (it) progresses silently until very advanced stage of liver disease is reached… if someone is otherwise well… you need to consider the risk versus the benefit of undertaking a procedure… if the stage of disease is unclear or the ethology of disease is unclear and a liver biopsy is needed, that’s quite an invasive test which carries a potential morbidity and even mortality.” Clinican3*.

Participants noted that there were differences in the treatment options available for chronic diseases in terms of their availability and effectiveness and that this may contribute to consumer motivation for screening, so was added as another attribute.

*“Firstly, screening Hepatitis C, fantastic, because we have a treatment and that treatment’s highly efficacious, it works, it’s easy to take, and so…. That gets over the barriers to implementing this sort of screening programme… NAFLD is a different kettle of fish. … at the moment we don’t have an effective treatment for NAFLD, other than weight loss and exercise, and if it was that easy, we wouldn’t have a problem of NAFLD and diabetes in the first place.“ Clinician1*.

*“A lot of people that get diagnosed with Hepatitis think it’s a death sentence…you’d be surprised how many didn’t even know… a simple tablet can cure you in days” Consumer2*.

The fourth theme centred around the screening test and process. The attributes discussed were the quality of the screening test from an accuracy and consistency perspective, and the waiting time for results.

*“there’s lots and lots of data now that we can believe in [specific screening test] results, as long as it’s done properly by a trained operator in a patient who’s fasted and things like that, then we have a lot of faith in the numbers” Clinician1*.

*“You want your results as soon as you can. Because otherwise you stress about it.” Clinician3*.

*“When you think breast screening… that brings a little bit of anxiety… I’ve had a lot of investigations done… and you do the test, and you wait for like 24 hours and you’re kind of like ’oh my gosh – that’s the end of me’, and then it’s ok.” Clinician4*.

The final theme focused on the broader system level context in which screening takes place and barriers that needed addressing in future screening programmes. Clinicians noted current system limitations including a lack of a national registry or centralised database to access historical scans, monitor trends, or enable better population health decision making, therefore this was an attribute that was included for consideration as part of a future screening programme.

“Probably an important thing that some central repository where everyone, every clinic, would be able to access the images for a particular patient. Like someone has got a little nodule... (is it) the same size as six months later or a year later?”

“We can’t measure easily who is getting screened (for liver disease) and who is not...you can certainly get that on breast screening and cervical cancer screening…”

“There is no registry... There is no Medicare item specifically for that… who’s getting – where is the gap?”

Another current barrier noted by participants related to the quality of scans and tests results from community settings. As such, an attribute around staff training and knowledge on the condition and screening process to be implemented was included.

“I guess one of the problems – so that at the moment there’s quite a spectrum of quality, of imaging, in the community.”

### Prioritisation exercise

Participants included consumers (n = 21), healthcare providers (n = 8) and one respondent who was categorised as both a consumer and a healthcare provider. The final prioritisation results of the 15 identified attributes were primarily determined by consumers (n = 22) (Table 3). Attributes with the lowest average score are deemed more important. Prioritisation results accounting for the views of both consumers and healthcare providers considered (i) ease of making an appointment (ii) accuracy and consistency of the test and (iii) information about a screening programme coming from a trusted source to be the three most important attributes.

**Table 3:** Final prioritisation exercise scores from consumers and clinicians combined

Score	Rank	Attribute
4.97	1	Ease of making an appointment to be screened
5.47	2	Quality of the test and results (accuracy, consistency)
6.20	3	Information on screening process and/ or value of being screened, comes from a trusted source
6.27	4	Patient receives a reminder or prompt to undertake screening
6.53	5	Positive patient experience with staff (i.e. friendly, culturally safe, non-judgemental)
6.87	6	Staff are trained and knowledgeable about the condition and screening process
7.00	7	Screening is integrated into a routine care appointment
7.17	8	Travel distance to screening location
7.57	9	Out-of-pocket costs for the patient
9.27	10	Availability and effectiveness of treatment options
9.30	11	Severity of the condition, current symptoms, and a patient’s other co-morbidities/ conditions
9.37	12	Physical experience of pain/ discomfort during screening procedure
9.93	13	Waiting time for results
12.03	14	Likelihood that additional testing/ invasive testing is required
12.07	15	Screening data is part of a registry to inform population health decisions (e.g. where to put more services)

Notably, when considering only consumer rankings (Additional File 3), similar outcomes were observed with the highest rank for: ‘information about a screening programme coming from a trusted source’. This was not a surprising difference between the overall results, given the much higher proportion of consumers in the prioritisation exercise. Further practical considerations such as ease of making an appointment and receiving a reminder were ranked highly by consumers.

When examining the healthcare provider results alone, accuracy and consistency of the test was ranked highest, with practical patient-related considerations such as ease of making an appointment, positive patient experience, travel time, and ‘out-of-pocket costs for the patient’ completing the top five. Interestingly, out of pocket costs for the patient ranked much higher in healthcare provider only rankings (Additional File 4) than in the consumer-only rankings.

### Expert panel

Results from the prioritisation exercise were used to inform expert panel discussions to determine a much smaller list of attributes (Table 4), with associated levels. It was determined that a maximum of five attributes should be taken forward from the original list of 15 to manage the cognitive burden for the participants [39]. The top five in each of the three lists were the starting point of the discussion. Participants discussed how different stakeholders prioritised the attributes, noting differences between consumers and clinicians. Further, they considered these rankings within the context of attributes and associated clinical relevance and value levels to the final DCE survey.

**Table 4:** Attributes and levels for inclusion in the DCE

Attribute	Level 1	Level 2	Level 3
Screening conduct	Nurse at local community health clinic	GP at your usual GP clinic	Specialist in hospital outpatient clinic
Quality and accuracy of the test results	85% accurate - For every 100 people who had a negative result, 15 would be incorrect and should have been positive	90% accurate - For every 100 people who had a negative result, 10 would be incorrect and should have been positive	95% accurate - For every 100 people who had a negative result, 5 would be incorrect and should have been positive
Cost to the patient/ consumer	$0 *(includes out of pocket costs such as parking, as well as lost income for time taken to undertake screening appointment)*	$80 *(includes out of pocket costs such as parking, as well as lost income for time taken to undertake screening appointment)*	$250 *(includes out of pocket costs such as parking, as well as lost income for time taken to undertake screening appointment)*
Wait time to appointment for screening	2 weeks	2 months	6 months
Source of information about importance of screening and screening process	Screening information is detailed and comes from a well trusted source *e.g. community member/ health professional you have a good relationship with discusses screening with you and provides a detailed flyer*	Screening information comes from a source which you would have a moderate amount of trust *e.g. community member/ health professional that you know moderately well quickly tells you that you need to be screened and hands you a short flyer*	Screening information is sent to you from a source where you have limited familiarity or trust *e.g. you receive a generic text, email or letter about screening*

The accuracy of the screening test was ranked highly in all three lists, and the panel agreed this was a key consideration for any screening programme. Ease of making an appointment, reminders, travel time, knowledgeable staff, and consumer experience were all highly rated, but after some discussion it was decided that these factors could be distilled into two key concepts – timeliness and the screening experience. As such, wait time for an appointment and screening conduct - a combination of who does the test and where - were agreed upon as amalgamated attributes. Further, these two aligned with the LOCATE-NAFLD care model which includes earlier access to liver screening thus relevant to health service decision makers.

Out of pocket costs were ranked 9th by consumers and 4th by health professionals but given that cost was emphasised as a barrier by all the focus group sessions, it was included in the proposed DCE design. Trusted source of information was ranked first by consumers, and despite this not being perceived as a priority by clinicians, this attribute was included in the proposed DCE design.

Panel participants noted that other attributes, like test invasiveness, pain or discomfort, or symptoms and other related co-morbidities were likely to have an impact on consumer desire to be screened. However, as these had not rated as highly, and considering the non-invasive screening test, these were not considered to be a priority over any of the attributes already discussed. Finalisation of exact wording of attributes and subsequent discussion on levels was completed via email with all participants and chief investigators, with a focus on clinical relevance and potential screening options. The final attributes and levels are in Table 4.

## Discussion

Five attributes with three levels were generated for the future DCE survey to elicit preferences for a community screening of liver disease. Best practice in the delivery of comprehensive models of care for NAFLD and other chronic liver diseases [40] is in evolution. Examples of strategies under evaluation include screening and fibrosis risk stratification with non-invasive tests, active management of patients in primary care to prevent disease progression, provision of integrated and coordinated care, and a multidisciplinary approach with provision of co-located services. The findings from this study provide further support for these strategies and the approach to implementation from the perspective of key stakeholders, health care consumers, clinicians, and health care managers.

Whilst there is limited literature on preferences for liver disease screening in community settings, other DCE studies for community screening programs for cancer have included similar attributes to that found in this study including those relating to the screening test or the health service delivery such as waiting time [41] and out-of-pocket costs [42]. In addition, two systematic reviews, conducted on DCEs for colorectal cancer [43], and DCEs for breast, colorectal and cervical cancers [41], both found that screening test effectiveness is the one attribute that is consistently found across all included studies, and our study is no different in that regard.

Lack of awareness is a major barrier to successfully implementing screening programmes. As highlighted during the focus group sessions, a lack of awareness about liver disease, the need for screening, or information on the test itself would have to be considered important issues to be addressed before implementing the screening programme widely. Engaging with other stakeholders beyond health, such as community groups, sports teams, and trusted sources of information, could address this issue as suggested by the focus group participants and in relevant literature [40]. The majority of NAFLD patients do not require intensive, specialist hepatology care to manage the disease at the initial stages [40]. Hence, raising awareness among primary health care providers about liver disease screening and management, as noted by study participants is another area to be considered prior to widespread implementation of the screening programme.

Community-based services in Australia have historically been cost-effective compared to secondary hospital care, particularly for chronic conditions [44]. Overall uptake of chronic disease screening programmes has previously been shown to be sub-optimal, so careful programme design considering factors that consumers value is needed [45]. Concerns about social stigmatisation regarding assumptions about lifestyle and cultural awareness were prominent themes discussed by patient advocates during the focus groups. This observation is particularly significant because it was often discussed in the context of other attributes such as health worker interactions and information and awareness, and so is perhaps a more deeply rooted theme for consideration. Other studies that have more closely examined stigmatisation in liver disease have shown that the association with alcohol causes cirrhotic patients to encounter discriminative attitudes, and patients tend to emotionally distance themselves from health services as a result [46, 47]. With respect to NAFLD, this issue is heightened as stigmatisation can also be driven by negative attitudes towards weight and obesity [48].

The main strength of this study is the inclusion of several different stakeholders including consumers in the focus group stage, which enabled identification of attributes that were both feasible and meaningful to those undertaking potential future screening programmes. Further, prioritisation and expert panel discussions allowed the broad range of views from the focus groups to be examined more deeply in terms of relative importance and focused into a more concise attribute selection for DCE choice sets that are plausible, tradable, and relevant for decision makers [32].

A key conclusion from this stage of the analysis was that attributes associated with each defined stage of a screening process; from initial awareness of the health issue and messaging, through to the process of booking and attending an appointment, the screening test itself and associated conduct, through to post-screening follow-up and treatment, are crucial to consider when designing choice sets for preference elicitation. Developing the DCE around the point of testing only may fail to recognise the broader reasons why people choose to, or fail to, attend health screening services. Additionally, it is critical to understand the attributes of care that cater to individual needs of each patient effectively for the successful implementation of the screening programme at the community level [40]. Hence, this study reports practical and feasible approaches to develop attributes to be included in the DCE study to elicit preferences for screening programmes for chronic liver diseases, as well as considerations for programme designers and implementers, which should in turn facilitate better policy decisions and implementation of chronic liver disease screening programmes in real world settings.

Limitations.

Due to resource and time limitations, the study included only participants who could communicate in English. Aboriginal and Torres Strait Islander peoples and other consumers from the most socioeconomically disadvantaged areas are overrepresented among patients with advanced chronic liver disease [49]. Whilst important insight was gained from the participant in the focus groups who identified as an Aboriginal, we recognise that this does not represent the range of First Nations cultural groups or perspectives. The barriers and limitations associated with the implementation of chronic liver disease screening programmes among different cultural and linguistically diverse groups should also be addressed in future research. It is also possible that preferences may differ according to the patient’s liver disease aetiology, or the screening test proposed. However, our focus was on creating a list of attributes and levels that can be used to elicit preferences for screening programmes across a range of chronic liver diseases.

## Conclusions

This mixed method study generated five key attributes for a future DCE on liver screening to inform better programme design and implementation. This paper describes the attribute development process in sufficient detail for replication and assessment of rigour. Importantly, the information reported highlights some broader social issues such as the stigma around liver disease that require careful consideration by policy makers.

### Electronic supplementary material

Below is the link to the electronic supplementary material.

Supplementary Material 1

Supplementary Material 2

Supplementary Material 3

Supplementary Material 4

## Data Availability

Recordings or transcripts of the focus groups are not publicly available as this was not approved within the ethics application and participant informed consent process, to preserve anonymity. All other data generated or analysed during this study are included in this published article and its additional files, except raw data from the prioritisation exercise which is available from the corresponding author on reasonable request.

## Abbreviations

DCE: Discrete choice experiment
LOCATE-NAFLD: LOCal Assessment and Triage Evaluation of Non-Alcoholic Fatty Liver Disease
NAFLD: Non-alcoholic fatty liver disease
QoL: Quality of life
