# Supplementary material for: A discrete choice experiment to elicit preferences for a liver screening programme in Queensland, Australia: a mixed methods study to select attributes and levels
Source: BMC Health Serv Res. 2023 Sep 5;23:950. doi: 10.1186/s12913-023-09934-2 (PMC10481473; doi:10.1186/s12913-023-09934-2)
Supplement: Supplementary file 1 — Supplementary Material 1 [file 12913_2023_9934_MOESM1_ESM.docx]

# Supplementary file 1

## Description

This supplementary table is a list of potential attributes for a DCE of community screening programme that resulted from the systematic review completed (Stage 1)

**S table 1: Potential attributes for a community screening DCE - stage 1**

|  | Action required by you personally to arrange test |
| --- | --- |
|  | Availability follow up test |
|  | Changes to organ(s) such as breast |
|  | Cognitive function |
|  | Co-morbidity |
|  | Cost of your test to a national health system |
|  | Discomfort after procedure |
|  | Discomfort during procedure |
|  | Effectiveness |
|  | False negative |
|  | False positive |
|  | Financial incentive |
|  | Frequency of procedure |
|  | Functional status |
|  | Generation of knowledge and effectiveness |
|  | Healthcare provider recommendation |
|  | Identification of population at risk |
|  | Listing of patients |
|  | Location |
|  | Maximization of follow up and treatment |
|  | Maximization of uptake |
|  | Method of screening |
|  | Mortality |
|  | Nature of procedure |
|  | Number of procedures |
|  | Nurse assistance prior to appointment |
|  | Operation of the program |
|  | Out of pocket cost for follow up care |
|  | Out of pocket cost for screening procedure |
|  | Overdiagnosis |
|  | Patient's age |
|  | Practitioner's sex |
|  | Preparation for procedure |
|  | Procedure information given prior to test |
|  | Procedure options |
|  | Procedure reminder |
|  | Procedure sensitivity |
|  | Procedure specificity |
|  | Reduction in mortality |
|  | Risk of later regret |
|  | Risk of overtreatment |
|  | Risk of unnecessary biopsy |
|  | Risk of unnecessary treatment |
|  | Scientific evidence |
|  | Screening duration |
|  | Screening interval |
|  | Sexual changes after procedure |
|  | Side effects of procedure |
|  | Staff assistance |
|  | Stakeholder's acting |
|  | Survival rate |
|  | Target population |
|  | Training for healthcare providers |
|  | Travel time |
|  | Waiting time for the screening procedure |
|  | Waiting time for the follow up test |
|  | Waiting time for the results |
|  | Who reviews the results |
