# Supplementary material for: A discrete choice experiment to elicit preferences for a liver screening programme in Queensland, Australia: a mixed methods study to select attributes and levels
Source: BMC Health Serv Res. 2023 Sep 5;23:950. doi: 10.1186/s12913-023-09934-2 (PMC10481473; doi:10.1186/s12913-023-09934-2)
Supplement: Supplementary file 2 — Supplementary Material 2 [file 12913_2023_9934_MOESM2_ESM.docx]

# Supplementary File 2

## S table 2: Semi-structured guide for focus groups or interviews

## Description:

Qualitative focus group guide – The following guide was used to conduct focus groups/interviews to elicit preferences for community-based screening programs.

## Objective:

The objective of the focus group/interview is to identify and explore potential attributes of community screening programs, and their associated levels (parameters), with key stakeholders.

## Participants:

Focus groups/interviews will be held with the following key groups as applicable:

- Healthcare providers (clinicians)
- Healthcare consumers

Maximum variation sampling should be used to ensure a variety of viewpoints are represented. Factors to consider may include area of responsibility, experience levels, geographic location and other socio-demographic features.

## How to use this guide:

The questions are based on a review of relevant literature, and relevant implementation science frameworks, to ensure all aspects of evaluation are considered in a comprehensive manner.

1. All key questions should be covered in the interview/focus group, however it is not necessary for interviewers to ask questions in the proposed order, or repeat a question if it has been answered in an earlier section
2. The conversation should explore issues as they are raised, with guidance and prompting as required
3. Facilitators will ensure discussion progresses in a timely, yet informative manner

## Introduction script:

*This section is included as an example of introductory text for the focus group/interview. Please note this can be adjusted and amended to suit the interviewer, context, format of delivery etc.*

Hi, and welcome to our focus group/interview exploring attributes of community screening programs. Thanks for giving us your time today. My name is ____________. I am a researcher with __________. The purpose of our discussion is to understand and explore the different attributes and preferences of effective, accessible and acceptable community health screening programs. Our discussion today will last about 90 minutes

This discussion that we wish to have with you today is part of a bigger project that is aimed at understanding how to maximise current health screening programs. As key stakeholders who make decisions about how community screening programs are designed and implemented at a system level, screening is undertaken and the information used from a clinical perspective, or how they are accessed and received from a consumer perspective, it is important that your views are taken into consideration when understanding the key attributes of a community screening program. We would like to find out your opinion, specifically, we will discuss the following issues with you:

- your experience with community screening programs
- attributes of current and ideal screening programs
- facilitators and barriers of community screening programs
- acceptable or expected levels/ parameters of these attributes

We are independent researchers, and this focus group/interview is confidential and voluntary, so please feel free to be as candid as you wish. Speaking of confidentiality, we do ask that you respect the focus group process and refrain from speaking about what was discussed in the focus group, after the session has been completed. We will record the conversation with you all if that is OK, but we will only share your conversation with other members of our research team or by making sure other people cannot identify your responses as an individual.

We have obtained ethical clearance for this study from _______________.

We really appreciate your time and willingness to speak with us. Does anyone/do you have any questions before we get started?

## Questions:

1. **Tell me about your experience of a community screening program/s?**

Prompts:

- *Which screening program was it?*
- *What was your role – manager/ clinician/ consumer?*

1. **What were some positive attributes of that screening program?**

Prompts:

- *Accessibility? Reach/Scale?*
- *Effectiveness? Performance/dependability? Limited false neg/ false pos?*
- *Acceptability? Ease of test/ no discomfort?*
- *Alignment with patient/clinician needs?*

1. **What are some of the things that facilitated that screening program?**

Prompts:

- *Political support?*
- *Integration with other systems?*
- *Coordinated implementation/roll-out/support?*
- *Good online resources/ marketing campaign?*
- *Consumer/clinician perceptions?*
- *Cost? Financial incentives?*

1. **What wasn’t so good about that screening program?**

Prompts:

- *What could be improved?*
- *Any feedback on consumer or clinician experience?*
- *Consider changes to accessibility, performance/dependability of test, difficulty/discomfort with the test itself, side effects, eligibility, risk of overtreatment?*

1. **What were some of the barriers to that screening program?**

*Prompts:*

- *What limits the uptake?*
- *What could be improved?*
- *Consider changes to information provided, coordination, wait times, changes to the clinician/ consumer interface, integration with other systems, better implementation/roll-out/support?*

1. **What would make an ideal community screening program?**

Prompts:

- Multiple perspectives -consumer, clinician, broader system/public health
- *Accessibility – Location, eligibility, wait times, Who conducts test, referral pathways, eligibility/expected cohort alignment, wait times, cost (individual or system),*
- *Acceptability - Ease of test, limited discomfort/side effects/invasiveness, Good support/ information, limited action required to arrange test, ability to engage consumers, meets needs of consumers and clinicians, evidence exists that screening will improve population outcomes, has political support?*
- *Effectiveness – test effectiveness, limited false neg/pos, potential for over diagnosis, speed of results, follow up treatment?*
- *Consequences – how different results might effect you, seriousness, comorbidities, side-effects?*
- *System - able to be implemented in a range of settings/locations/population groups, structural elements required exist/ are in place?*

1. **We have listed here on the whiteboard/screen your suggestions for attributes from today’s session. Let’s explore the levels or parameters of these.**

Prompts:

*To what extent?*

*What level would/ wouldn’t be acceptable?*

*Maximum/ minimum?*

*What impact would that level/parameter have?*

1. **Is there anything else you wanted to say about community screening programs and your experience with it?**

End of guide
