# Supplementary material for: A discrete choice experiment to elicit preferences for a liver screening programme in Queensland, Australia: a mixed methods study to select attributes and levels
Source: BMC Health Serv Res. 2023 Sep 5;23:950. doi: 10.1186/s12913-023-09934-2 (PMC10481473; doi:10.1186/s12913-023-09934-2)
Supplement: Supplementary file 3 — Supplementary Material 3 [file 12913_2023_9934_MOESM3_ESM.docx]

# Supplementary File 3

Description

Supplementary file 3 contains the results from the prioritisation exercise for the consumer responses only.

**S table 3: Final prioritisation exercise scores from consumers only**

| **Score** | **Rank** | **Attribute** |
| --- | --- | --- |
| 5.05 | 1 | Information on screening process and/ or value of being screened, comes from a trusted source |
| 5.14 | 2 | Ease of making an appointment to be screened |
| 5.55 | 3 | Quality of the test and results (accuracy, consistency) |
| 6.18 | 4 | Patient receives a reminder or prompt to undertake screening |
| 6.27 | 5 | Staff are trained and knowledgeable about the condition and screening process |
| 7.09 | 6 | Screening is integrated into a routine care appointment |
| 7.45 | 7 | Travel distance to screening location |
| 7.50 | 8 | Positive patient experience with staff (i.e. friendly, culturally safe, non-judgemental) |
| 7.91 | 9 | Out-of-pocket costs for the patient |
| 8.95 | 10 | Severity of the condition, current symptoms, and a patient’s other co-morbidities/ conditions |
| 9.32 | 11 | Waiting time for results |
| 9.82 | 12 | Availability and effectiveness of treatment options |
| 10.32 | 13 | Physical experience of pain/ discomfort during screening procedure |
| 11.50 | 14 | Likelihood that additional testing/ invasive testing is required |
| 11.95 | 15 | Screening data is part of a registry to inform population health decisions (e.g. where to put more services) |
