# Supplementary material for: A discrete choice experiment to elicit preferences for a liver screening programme in Queensland, Australia: a mixed methods study to select attributes and levels
Source: BMC Health Serv Res. 2023 Sep 5;23:950. doi: 10.1186/s12913-023-09934-2 (PMC10481473; doi:10.1186/s12913-023-09934-2)
Supplement: Supplementary file 4 — Supplementary Material 4 [file 12913_2023_9934_MOESM4_ESM.docx]

# Supplementary File 4

Description

Supplementary File 4 contains the results from the prioritisation exercise for the clinician responses only.

**S table 4: Final prioritisation exercise scores from consumers only**

| **Score** | **Rank** | **Attribute** |
| --- | --- | --- |
| 4.89 | 1 | Quality of the test and results (accuracy, consistency) |
| 5.00 | 2 | Ease of making an appointment to be screened |
| 5.22 | 3 | Positive patient experience with staff (i.e. friendly, culturally safe, non-judgemental) |
| 6.44 | 4 | Out-of-pocket costs for the patient |
| 6.78 | 5 | Travel distance to screening location |
| 6.89 | 6 | Staff are trained and knowledgeable about the condition and screening process |
| 7.00 | 7 | Availability and effectiveness of treatment options |
| 7.11 | 8 | Patient receives a reminder or prompt to undertake screening |
| 7.44 | 9 | Screening is integrated into a routine care appointment |
| 7.56 | 10 | Physical experience of pain/ discomfort during screening procedure |
| 8.67 | 11 | Information on screening process and/ or value of being screened, comes from a trusted source |
| 10.00 | 12 | Severity of the condition, current symptoms, and a patient’s other co-morbidities/ conditions |
| 11.56 | 13 | Waiting time for results |
| 11.78 | 14 | Screening data is part of a registry to inform population health decisions (e.g. where to put more services) |
| 13.67 | 15 | Likelihood that additional testing/ invasive testing is required |
